# Pomalidomide, bortezomib, and dexamethasone for multiple myeloma previously treated with lenalidomide (OPTIMISMM): outcomes by prior treatment at first relapse

**DOI:** 10.1038/s41375-020-01021-3

**Published:** 2020-09-07

**Authors:** Meletios Dimopoulos, Katja Weisel, Philippe Moreau, Larry D. Anderson, Darrell White, Jesus San-Miguel, Pieter Sonneveld, Monika Engelhardt, Matthew Jenner, Alessandro Corso, Jan Dürig, Michel Pavic, Morten Salomo, Eva Casal, Shankar Srinivasan, Xin Yu, Tuong Vi Nguyen, Tsvetan Biyukov, Teresa Peluso, Paul Richardson

**Affiliations:** 1 National and Kapodistrian University of Athens, Athens, Greece; 2 Department of Oncology, Hematology and Bone Marrow Transplantation with Section of Pneumology, University Medical Center Hamburg-Eppendorf, Hamburg, Germany; 3 University Hospital Hôtel-Dieu, Nantes, France; 4 The University of Texas Southwestern Medical Center, Dallas, TX USA; 5 Dalhousie University and Queen Elizabeth II Health Sciences Centre, Halifax, NS Canada; 6 Clinica Universidad de Navarra, CIMA, IDISNA, Pamplona, Spain; 7 Erasmus MC Cancer Institute, Rotterdam, The Netherlands; 8 Faculty of Freiburg, Universitätsklinikum Freiburg, Freiburg, Germany; 9 University Hospital Southampton, Southampton, UK; 10 Division of Hematology, Hospital Legnano, Milan, Italy; 11 University Medicine Essen, Essen, Germany; 12 Centre Hospitalier Universitaire De Sherbrooke (CHUS) - Centre de Recherche Clinique Etienne-Le Bel (CRCELB) Hôpital Fleurimont, Sherbrooke, QC Canada; 13 Copenhagen University Hospital, Rigshospitalet, Copenhagen Denmark; 14 Bristol Myers Squibb, Summit, NJ USA; 15 Celgene International Sàrl, Bristol Myers Squibb Company, Boudry, Switzerland; 16 Department of Medical Oncology, Jerome Lipper Multiple Myeloma Center, Dana-Farber Cancer Institute, Harvard Medical School, Boston, MA USA

**Keywords:** Myeloma, Randomized controlled trials

## Abstract

In the phase 3 OPTIMISMM trial, pomalidomide, bortezomib, and dexamethasone (PVd) demonstrated superior efficacy vs bortezomib and dexamethasone (Vd) in patients with relapsed or refractory multiple myeloma previously treated with lenalidomide, including those refractory to lenalidomide. This analysis evaluated outcomes in patients at first relapse (*N* = 226) by lenalidomide-refractory status, prior bortezomib exposure, and prior stem cell transplant (SCT). Second-line PVd significantly improved PFS vs Vd in lenalidomide-refractory (17.8 vs 9.5 months; *P* = 0.0276) and lenalidomide-nonrefractory patients (22.0 vs 12.0 months; *P* = 0.0491), patients with prior bortezomib (17.8 vs 12.0 months; *P* = 0.0068), and patients with (22.0 vs 13.8 months; *P* = 0.0241) or without (16.5 vs 9.5 months; *P* = 0.0454) prior SCT. In patients without prior bortezomib, median PFS was 20.7 vs 9.5 months (*P* = 0.1055). Significant improvement in overall response rate was also observed with PVd vs Vd in lenalidomide-refractory (85.9% vs 50.8%; *P* < 0.001) and lenalidomide-nonrefractory (95.7% vs 60.0%; *P* < 0.001) patients, with similar results regardless of prior bortezomib or SCT. No new safety signals were observed. These data demonstrate the benefit of PVd at first relapse, including immediately after upfront lenalidomide treatment failure and other common first-line treatments.

## Introduction

Lenalidomide, an oral immunomodulatory agent, is an established standard of care for patients with newly diagnosed multiple myeloma (NDMM) [1]. Because frontline lenalidomide is routinely used until disease progression, most patients will become refractory to lenalidomide early in their treatment course and represent a clinically important patient population in need of proven therapies [2]. Moreover, with successive relapses, outcomes worsen and the interval between relapses shortens, underscoring the need for effective therapies in early treatment lines to maximize response and delay progression [3–7].

Pomalidomide, an oral immunomodulatory agent like lenalidomide, exerts potent, direct tumoricidal and immune-enhancing effects [8]. Although pomalidomide and lenalidomide belong to the same drug class, pomalidomide has exhibited antitumor and immunomodulatory activity in lenalidomide-resistant cell lines and animal models [9–11]. The combination of pomalidomide and dexamethasone is a standard treatment option for patients with relapsed or refractory multiple myeloma (RRMM) previously treated with lenalidomide, and pomalidomide has demonstrated a survival benefit in lenalidomide-refractory disease; moreover, it is the only agent that has been extensively studied in the post-lenalidomide treatment setting [2, 12–18].

Pomalidomide has demonstrated synergistic antimyeloma activity with dexamethasone and other agents, including proteasome inhibitors and monoclonal antibodies, even after lenalidomide-based therapy [11, 19–24]. As a result, triplet regimens based on the pomalidomide-and-dexamethasone doublet have recently gained regulatory approval for the treatment of patients with RRMM, with agents such as daratumumab and isatuximab in the United States, elotuzumab in the United States and European Union, and bortezomib in the European Union and other countries [25–29].

The approval of pomalidomide, bortezomib, and dexamethasone (PVd) was based on results from the phase 3 OPTIMISMM trial in lenalidomide-pretreated patients with early-line RRMM (70% lenalidomide refractory; median prior lines of therapy, 2) [16, 25]. PVd significantly improved progression-free survival (PFS) compared with bortezomib and dexamethasone (Vd; median, 11.20 vs 7.10 months; hazard ratio [HR], 0.61 [95% CI, 0.49–0.77]; *P* < 0.0001). The safety profile of PVd aligned with the known toxicity profiles of the constituent agents.

Given the need for proven therapies following upfront lenalidomide treatment, we performed a post hoc subanalysis of the OPTIMISMM trial to evaluate the efficacy and safety of PVd vs Vd in patients at first relapse (i.e, after only 1 prior line of therapy). Patients were evaluated according to features representative of real-world patients, including lenalidomide-refractory status, prior exposure to bortezomib, and prior stem cell transplant (SCT).

## Materials and methods

### Patients and study design

Details of the OPTIMISMM trial have been previously reported by Richardson et al. [16]. Briefly, OPTIMISMM is an international, randomized, open-label, controlled, phase 3 clinical trial (NCT01734928). Eligible patients were aged ≥18 years and had a diagnosis of multiple myeloma, measurable disease, and an Eastern Cooperative Oncology Group performance status of 0–2. Patients were required to have had 1–3 prior treatment regimens (including ≥2 cycles of lenalidomide) and progressive disease (PD) during or after their last antimyeloma regimen. Key exclusion criteria included creatinine clearance <30 mL/min requiring dialysis, grade ≥3 peripheral neuropathy, or grade 2 peripheral neuropathy with pain. Patients who were refractory to lenalidomide were eligible, including those who received lenalidomide in their last prior regimen. Refractory patients were defined as patients with disease that was nonresponsive to treatment (failure to achieve minimum response) or who developed PD within 60 days of the last dose, inclusive. Patients with prior exposure to bortezomib were eligible, provided they were not refractory to a bortezomib-containing regimen dosed at 1.3 mg/m^2^ twice weekly. Written informed consent was obtained from all patients. The study adhered to the principles of Good Clinical Practice according to the International Conference on Harmonisation requirements and the Declaration of Helsinki, and the study protocol was approved by the institutional review board or central or local ethics committee at each participating study site.

### Treatment

Patients were randomized 1:1 to receive PVd or Vd. Treatment was administered in 21-day cycles until PD or unacceptable toxicity. Patients received bortezomib 1.3 mg/m^2^ on days 1, 4, 8, and 11 of cycles 1–8 and on days 1 and 8 of cycles 9 and beyond. Dexamethasone was given on days 1, 2, 4, 5, 8, 9, 11, and 12 of cycles 1–8 and on days 1, 2, 8, and 9 of cycles 9 and beyond; patients received 20 mg of dexamethasone if aged ≤75 years and 10 mg otherwise. Patients in the PVd arm received pomalidomide 4 mg on days 1–14 of each cycle.

### Endpoints and assessments

The primary endpoint was PFS. Secondary endpoints were overall survival, overall response rate (ORR), duration of response, and safety. Data were not mature for the planned interim analysis of overall survival (data cutoff, October 26, 2017). Time to response was an exploratory endpoint. The Kaplan–Meier method was used to estimate PFS. ORR was assessed by the International Myeloma Working Group criteria. Primary, secondary, and exploratory analyses were conducted in the intention-to-treat population, which included all randomized patients. Safety analyses were conducted in the safety population, which was composed of all patients who received ≥1 dose of study medication. SAS software (version 9.2) was used for statistical analysis. Efficacy subgroup analyses were not adjusted by stratification factors.

## Results

### Patients

Overall, 226 of the 559 patients in the intention-to-treat population of OPTIMISMM had only 1 prior line of therapy (Table 1; Supplemental Fig. 1). Of these patients, 129 (57.1%) were refractory to lenalidomide (PVd arm, 64 patients; Vd arm, 65 patients), and 97 (42.9%) were nonrefractory to lenalidomide (PVd arm, 47 patients; Vd arm, 50 patients). The median age was 69.0 years for patients refractory to lenalidomide and 66.0 years for patients nonrefractory to lenalidomide. The lenalidomide-refractory subgroup had a lower proportion of patients with prior SCT vs the lenalidomide-nonrefractory subgroup (38.8% vs 61.9%). Baseline characteristics were generally balanced between the 2 treatment arms in each subgroup. However, a larger proportion of patients had impaired renal function (creatinine clearance < 60 mL/min) and high-risk cytogenetics, and a smaller proportion of patients were International Staging System stage III in the PVd arm compared with the Vd arm of the lenalidomide-refractory subgroup. Similarly, a larger proportion of patients had impaired renal function in the PVd vs Vd arms of the no-prior-bortezomib and no-prior-SCT patient subgroups (Supplemental Table 1).

**Table 1:** Baseline characteristics of patients at first relapse by lenalidomide-refractory status.

Characteristic	Patients at first relapse^a^			
	LEN refractory		LEN nonrefractory	
	PVd (*n* = 64)	Vd (*n* = 65)	PVd (*n* = 47)	Vd (*n* = 50)
Age, median (range), years	68 (38–87)	69 (27–89)	66 (29–81)	66 (41–84)
>65 years, *n* (%)	37 (57.8)	39 (60.0)	25 (53.2)	25 (50.0)
>75 years, *n* (%)	7 (10.9)	9 (13.8)	9 (19.1)	9 (18.0)
Male, *n* (%)	37 (57.8)	38 (58.5)	30 (63.8)	19 (38.0)
ECOG PS, *n* (%)				
0	36 (56.3)	33 (50.8)	31 (66.0)	26 (52.0)
1	26 (40.6)	27 (41.5)	15 (31.9)	22 (44.0)
2	2 (3.1)	5 (7.7)	1 (2.1)	2 (4.0)
ISS stage, *n* (%)				
I	38 (59.4)	40 (61.5)	27 (57.4)	29 (58.0)
II	19 (29.7)	13 (20.0)	14 (29.8)	14 (28.0)
III	7 (10.9)	12 (18.5)	6 (12.8)	7 (14.0)
Cytogenetic profile by FISH, *n* (%)^b^				
Standard risk	33 (51.6)	30 (46.2)	25 (53.2)	26 (52.0)
High risk	13 (20.3)	8 (12.3)	5 (10.6)	6 (12.0)
Missing or NE	18 (28.1)	27 (41.5)	17 (36.2)	18 (36.0)
Time since MM diagnosis, median (range), years	2.7 (0.2–10.8)	2.6 (0.4–11.1)	3.1 (0.9–7.6)	4.0 (0.6–12.8)
Creatinine clearance < 60 mL/min, *n* (%)	24 (37.5)	16 (24.6)	11 (23.4)	12 (24.0)
Prior antimyeloma lines of therapy, median (range)	1 (1–1)	1 (1–1)	1 (1–1)	1 (1–1)
Previous treatment, *n* (%)				
Lenalidomide	64 (100)	65 (100)	47 (100)	50 (100)
Bortezomib	36 (56.3)	31 (47.7)	31 (66.0)	36 (72.0)
Stem cell transplant	26 (40.6)	24 (36.9)	30 (63.8)	30 (60.0)
Refractory status, *n* (%)^c^				
Lenalidomide	64 (100)	65 (100)	0	0
Bortezomib	6 (9.4)	6 (9.2)	5 (10.6)	1 (2.0)

*ECOG PS* Eastern Cooperative Oncology performance status, *FISH* fluorescence in situ hybridization, *ISS* International Staging System, *LEN* lenalidomide, *MM* multiple myeloma, *PD* progressive disease, *PVd* pomalidomide, bortezomib, and dexamethasone, *Vd* bortezomib plus dexamethasone.

^a^Patients with only 1 prior line of therapy.

^b^High risk was defined as the presence of ≥1 of the following cytogenetic abnormalities: del(17p) (including monosomy 17), t(4;14), and/or t(14;16). The standard risk was defined as the absence of high-risk cytogenetic abnormalities.

^c^Refractory disease was defined as a disease that was nonresponsive to treatment (failure to achieve minimum response or development of PD) within 60 days of the last dose, inclusive.

### Disposition and treatment exposure

At the time of data cutoff (October 26, 2017), 40 patients (31.0%) in the lenalidomide-refractory subgroup and 33 patients (34.0%) in the lenalidomide-nonrefractory subgroup remained on treatment, with the majority on PVd (Table 2). The most common cause of treatment discontinuation was PD. In both subgroups, duration of treatment was longer with PVd vs Vd (lenalidomide refractory: median, 9.7 vs 6.1 months; lenalidomide nonrefractory: median, 13.6 vs 6.6 months). Patients who received PVd had more treatment cycles than those who received Vd (lenalidomide refractory: median, 13 vs 9 cycles; lenalidomide nonrefractory: median, 18 vs 9 cycles).

**Table 2:** Patient disposition and treatment exposure of patients at first relapse by lenalidomide-refractory status.

	Patients at first relapse^a^			
	LEN refractory		LEN nonrefractory	
	ITT population			
Patient disposition, *n* (%)	PVd (*n* = 64)	Vd (*n* = 65)	PVd (*n* = 47)	Vd (*n* = 50)
Ongoing treatment	27 (42.2)	13 (20.0)	20 (42.6)	13 (26.0)
Discontinued treatment	37 (57.8)	49 (75.4)	27 (57.4)	35 (70.0)
Progressive disease	22 (34.4)	35 (53.8)	12 (25.5)	16 (32.0)
Adverse event	4 (6.3)	7 (10.8)	6 (12.8)	13 (26.0)
Withdrawal of consent	7 (10.9)	2 (3.1)	3 (6.4)	4 (8.0)
Death	2 (3.1)	1 (1.5)	4 (8.5)	0
Other	2 (3.1)	3 (4.6)	2 (4.3)	2 (4.0)
Pregnancy	0	1 (1.5)	0	0

	Safety population			
Treatment exposure	PVd (*n* = 64)	Vd (*n* = 62)	PVd (*n* = 47)	Vd (*n* = 48)
No treatment received, *n* (%)	0	3 (4.6)	0	2 (4.0)
Duration of treatment, median (range), months	9.7 (1.1–33.8)	6.1 (0.7–37.2)	13.6 (1.9–28.0)	6.6 (0.1–23.9)
No. of treatment cycles, median (range)	13 (2–46)	9 (1–53)	18 (3–40)	9 (1–35)

*ITT* intention-to-treat, *LEN* lenalidomide, *PVd* pomalidomide, bortezomib, and dexamethasone, *Vd* bortezomib plus dexamethasone.

^a^Patients with only 1 prior line of therapy.

### Efficacy

**Fig. 1:**
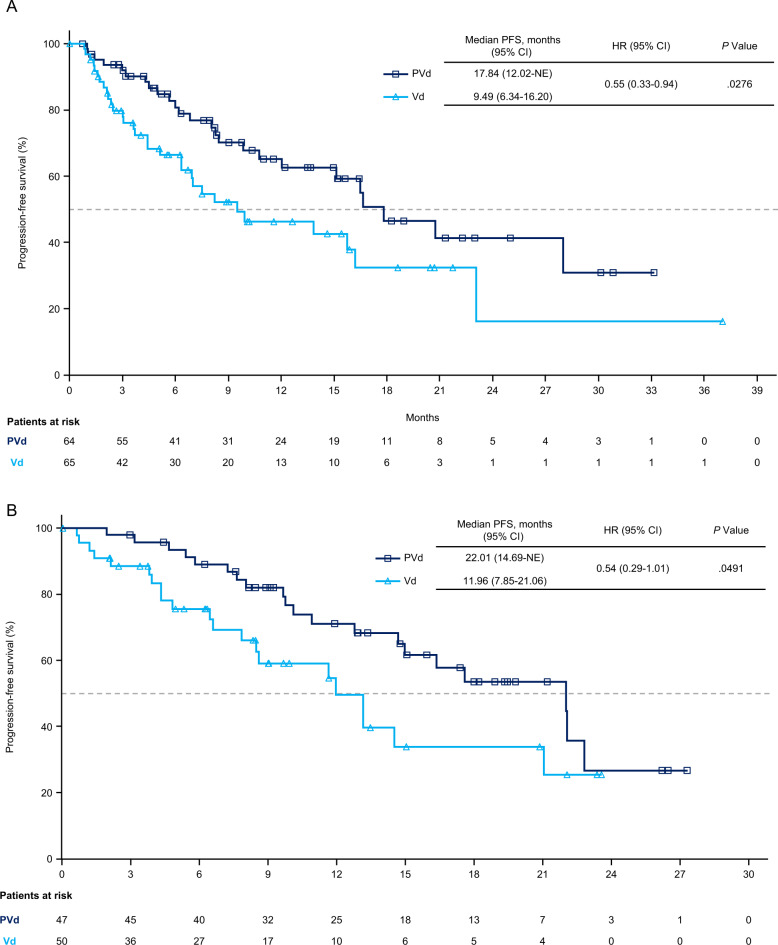
Progression-free survival in patients at first relapse (1 prior line of therapy) by lenalidomide-refractory status. **a** Patients who were refractory to lenalidomide at first relapse. **b** Patients who were nonrefractory to lenalidomide at first relapse. HR hazard ratio, NE not evaluable, PFS progression-free survival, PVd pomalidomide, bortezomib, and dexamethasone, Vd bortezomib plus dexamethasone.

In all patients treated with PVd at first relapse, PFS and ORR were significantly improved vs in those treated with Vd (PFS: median, 20.73 vs 11.63 months; HR, 0.54 [95% CI, 0.36–0.82]; *P* = 0.0027; previously reported [16]; ORR: 90.1 vs 54.8%; odds ratio [OR], 7.50 [95% CI, 3.64–15.46]; *P* < 0.001; Supplemental Table 2). Second-line PVd also led to significant improvements in PFS regardless of lenalidomide-refractory status. Median PFS was 17.8 months with PVd vs 9.5 months with Vd (HR, 0.55 [95% CI, 0.33–0.94]; *P* = 0.0276; Fig. 1a) in the lenalidomide-refractory subgroup and 22.0 vs 12.0 months (HR, 0.54 [95% CI, 0.29–1.01]; *P* = 0.0491), in the lenalidomide-nonrefractory subgroup (Fig. 1b); median follow-up was 16.4 months. ORR was also significantly improved with PVd vs Vd in lenalidomide-refractory patients (85.9% vs 50.8%; OR, 5.93 [95% CI, 2.52–13.95]; *P* < 0.001; Table 3) and in lenalidomide-nonrefractory patients (95.7% vs 60.0%; OR, 15.00 [95% CI, 3.26–68.94]; *P* < 0.001). The proportion of patients who achieved at least very good partial response in the lenalidomide-refractory subgroup was 56.3% in the PVd vs 23.1% in the Vd arm, and 68.1% vs 22.0% in the lenalidomide-nonrefractory subgroup. The median time to response was 1.4 months (range, 0.7–5.4 months) with PVd and 1.0 month (range, 0.7–6.2 months) with Vd in lenalidomide-refractory patients (*P* = 0.555) and 0.9 months (range, 0.7–3.0 months) with PVd and 1.4 months (range, 0.7–2.8 months) with Vd in lenalidomide-nonrefractory patients (*P* = 0.059). The median duration of response was 20.0 months with PVd and 14.8 months with Vd in patients who were refractory to lenalidomide, and 20.7 months with PVd and 13.8 months with Vd in patients who were nonrefractory to lenalidomide.

**Table 3:** Overall response rate in patients at first relapse by lenalidomide-refractory status.

	Patients at first relapse^a^			
	LEN refractory		LEN nonrefractory	
Response rates, *n* (%)	PVd (*n* = 64)	Vd (*n* = 65)	PVd (*n* = 47)	Vd (*n* = 50)
Overall response rate	55 (85.9)	33 (50.8)	45 (95.7)	30 (60.0)
≥VGPR	36 (56.3)	≥15 (23.1)	32 (68.1)	11 (22.0)
sCR	2 (3.1)	2 (3.1)	4 (8.5)	0
CR	6 (9.4)	3 (4.6)	8 (17.0)	2 (4.0)
VGPR	28 (43.8)	10 (15.4)	20 (42.6)	9 (18.0)
PR	19 (29.7)	18 (27.7)	13 (27.7)	19 (38.0)
SD	8 (12.5)	28 (43.1)	2 (4.3)	12 (24.0)
PD	1 (1.6)	1 (1.5)	0	3 (6.0)
NE	0	3 (4.6)	0	5 (10.0)

*CR* complete response, *LEN* lenalidomide, *NE* not evaluable, *PD* progressive disease, *PR* partial response, PVd pomalidomide, bortezomib, and dexamethasone, *sCR* stringent complete response, *SD* stable disease, *Vd* bortezomib plus dexamethasone, *VGPR* very good partial response.

^a^Patients with only 1 prior line of therapy.

A significant improvement in PFS was also observed with second-line PVd vs Vd in patients with prior exposure to bortezomib (median, 17.8 vs 12.0 months; HR, 0.47 [95% CI, 0.26–0.82]; *P* = 0.0068; Supplemental Fig. 2). Of note, median PFS values did not change after excluding bortezomib-refractory patients (11 patients [16.4%] in the PVd arm and 7 patients [10.4%] in the Vd arm); in OPTIMISMM, bortezomib-refractory patients were only eligible if they were not refractory to a bortezomib-containing regimen dosed at 1.3 mg/m^2^ twice weekly. In patients without prior exposure to bortezomib, median PFS was 20.7 months with PVd and 9.5 months with Vd (HR, 0.62 [95% CI, 0.35–1.11]; *P* = 0.1055). Among patients who were previously treated with lenalidomide, bortezomib, and dexamethasone (RVd; *n* = 48), a common first-line regimen, median PFS was 14.7 months with PVd (*n* = 20) and 11.6 months with Vd (*n* = 28; HR, 0.58 [95% CI, 0.26–1.31]; *P* = 0.1927). Furthermore, PVd significantly improved ORR in patients vs Vd, regardless of prior exposure to bortezomib (Supplemental Table 3). The ORR was 89.6% with PVd vs 49.3% with Vd in patients with prior bortezomib exposure (OR, 8.83 [95% CI, 3.53–22.11]; *P* < 0.001) and 90.9% with PVd vs 62.5% with Vd in patients without prior bortezomib exposure (OR, 6.00 [95% CI, 1.84–19.57]; *P* = 0.002). Efficacy outcomes were improved with PVd vs Vd regardless of prior SCT. PFS was significantly longer with PVd vs Vd in patients who underwent prior SCT (median, 22.0 vs 13.8 months; HR, 0.48 [95% CI, 0.25–0.92]; *P* = 0.0241; Supplemental Fig. 3), as well as in patients who did not undergo SCT (median, 16.5 vs 9.5 months; HR, 0.59 [95% CI, 0.35–0.99]; *P* = 0.0454). Patients who underwent prior SCT achieved an ORR of 91.1% with PVd vs 57.4% with Vd (OR, 7.57 [95% CI, 2.61–21.96]; *P* < 0.001). ORR was 89.1% with PVd vs 52.5% with Vd in patients who did not have prior SCT (OR, 7.40 [95% CI, 2.76–19.83]; *P* < 0.001).

### Safety

In the lenalidomide-refractory subgroup, 90.6% and 71.0% of patients who received PVd and Vd, respectively, experienced ≥1 grade 3/4 treatment-emergent adverse event (TEAE; Table 4); among lenalidomide-nonrefractory patients, the rate of grade 3/4 TEAEs was 85.1% with PVd and 64.6% with Vd. In general, rates of grade 3/4 TEAEs reported with PVd were similar in both subgroups of patients. The most common grade 3/4 hematologic TEAEs (PVd vs Vd) in lenalidomide-refractory patients included neutropenia (35.9% vs 12.9%) and thrombocytopenia (17.2% vs 22.6%). Grade 3/4 infections were the most frequently reported nonhematologic TEAE (29.7% vs 21.0%), including pneumonia (9.4% vs 9.7%); grade 3/4 peripheral sensory neuropathy was reported in 9.4% and 3.2% of patients, respectively. In lenalidomide-nonrefractory patients, grade 3/4 neutropenia and thrombocytopenia were reported in 36.2% and 23.4% of patients, respectively, who received PVd and 6.3% and 18.8% of patients who received Vd. Grade 3/4 peripheral sensory neuropathy was observed in 8.5% vs 4.2% of patients in the PVd vs Vd arms of the lenalidomide-nonrefractory subgroup. Grade 3/4 infections were reported in 27.7% vs 8.3% of lenalidomide-nonrefractory patients in the PVd vs Vd arms, including pneumonia, which was observed in 8.5% of patients who received PVd and no patients who received Vd.

**Table 4:** Grade 3/4 treatment-emergent adverse events in patients at first relapse by lenalidomide-refractory status.

	Patients at first relapse^a^			
	LEN refractory		LEN nonrefractory	
TEAEs, *n* (%)^b^	PVd (*n* = 64)	Vd (*n* = 62)	PVd (*n* = 47)	Vd (*n* = 48)
Patients with ≥ 1 grade 3/4 TEAE	58 (90.6)	44 (71.0)	40 (85.1)	31 (64.6)
Grade 3/4 hematologic TEAEs				
Neutropenia	23 (35.9)	8 (12.9)	17 (36.2)	3 (6.3)
Febrile neutropenia	2 (3.1)	0	1 (2.1)	0
Thrombocytopenia	11 (17.2)	14 (22.6)	11 (23.4)	9 (18.8)
Anemia	11 (17.2)	5 (8.1)	1 (2.1)	2 (4.2)
Grade 3/4 nonhematologic TEAEs				
Infections	19 (29.7)	13 (21.0)	13 (27.7)	4 (8.3)
Pneumonia	6 (9.4)	6 (9.7)	4 (8.5)	0
Hyperglycemia	7 (10.9)	7 (11.3)	2 (4.3)	2 (4.2)
Peripheral sensory neuropathy	6 (9.4)	2 (3.2)	4 (8.5)	2 (4.2)
Fatigue	4 (6.3)	2 (3.2)	5 (10.6)	1 (2.1)
Diarrhea	4 (6.3)	3 (4.8)	4 (8.5)	3 (6.3)
Rash	0	0	5 (10.6)	0

*LEN* lenalidomide, *PVd* pomalidomide, bortezomib, and dexamethasone, *TEAE* treatment-emergent adverse event, *Vd* bortezomib plus dexamethasone.

^a^Patients with only 1 prior line of therapy.

^b^Reported in ≥8% of patients in any treatment arm, except for febrile neutropenia.

Pomalidomide dose reductions and interruptions due to AEs in the PVd vs Vd arms were observed in 37.5% vs 64.1% of lenalidomide-refractory patients and 51.1% vs 89.4% of lenalidomide-nonrefractory patients. Bortezomib dose reductions due to AEs (PVd vs Vd) were reported in 51.6% vs 43.5% of lenalidomide-refractory patients and 59.6% vs 43.8% of lenalidomide-nonrefractory patients. In patients who were refractory to lenalidomide, bortezomib dose interruptions due to AEs (PVd vs Vd) were observed in 50.0% vs 61.3% of patients; in patients who were nonrefractory to lenalidomide, the rates were 59.6% vs 47.9%.

## Discussion

Despite great advances in antimyeloma pharmacotherapy, multiple myeloma remains incurable and nearly all patients relapse [3, 4, 30]. Treatment of patients with RRMM remains challenging due to a multitude of factors such as age, frailty, prior therapies, and the evolving complexity of therapeutic options [3, 31]. In particular, patients who become refractory to lenalidomide are a clinically relevant population, as lenalidomide is a standard of care in the treatment of NDMM [1, 2]. Because outcomes deteriorate with successive relapses, effective therapeutic intervention in early lines of RRMM is critical [5, 6]. Thus, lenalidomide-pretreated patients with RRMM need proven regimens early in their disease course.

To date, OPTIMISMM is the only phase 3 trial designed to address the treatment of patients with RRMM following early-line lenalidomide and, to our knowledge, the first to report efficacy outcomes by lenalidomide-refractory status in patients treated at first relapse. In this analysis, second-line treatment with PVd significantly reduced the risk of progression or death vs Vd by 45% in lenalidomide-refractory patients (*P* = 0.0276) and by 46% (*P* = 0.0491) in lenalidomide-nonrefractory patients. Furthermore, the addition of pomalidomide to Vd at first relapse resulted in significantly improved ORR vs Vd alone, regardless of refractory status to lenalidomide (*P* < 0.001 for both subgroups), and led to deeper and more durable responses. In both of these subgroups, treatment duration was longer and the number of treatment cycles received was larger with PVd vs Vd. Similar trends of improved efficacy outcomes with PVd at first relapse were observed, regardless of prior exposure to bortezomib or prior SCT. Additionally, among patients who were previously treated with RVd, median PFS was longer with PVd vs Vd (14.7 vs 11.6 months), although the difference for this post hoc comparison did not reach statistical significance (*P* = 0.1927). This is likely attributable to the small sample size for the prior RVd subgroup (*n* = 48), which reflects the treatment landscape when OPTIMISMM began patient accrual in 2012; the manuscript for the phase 3 SWOG S0777 RVd trial was not published until 2017 [32]. The safety profile of PVd at first relapse was generally consistent with the known safety profiles of pomalidomide, bortezomib, and dexamethasone. Taken together, our findings demonstrate the benefits of PVd at first relapse in patients for whom lenalidomide is no longer a treatment option, including patients refractory to lenalidomide. Moreover, given that 61.2% of patients who were refractory to lenalidomide did not have prior SCT, these data demonstrate the feasibility and efficacy of PVd in a patient population that likely has fewer proven treatment options following lenalidomide due to age or comorbidities that preclude the use of intensive therapies.

Importantly, these findings demonstrate that continued immunomodulation with pomalidomide-based therapy brings added benefit in successive treatment lines. The activity and efficacy of pomalidomide in lenalidomide-resistant disease is well established [9–16]. The phase 2 MM-014 trial (NCT01946477) investigated pomalidomide and dexamethasone (cohort A) and pomalidomide, dexamethasone, and daratumumab (cohort B) in patients with early-line RRMM, all of whom were exposed to lenalidomide in their immediate prior line of treatment. In cohort A, in which 87.5% of patients were refractory to their most recent prior lenalidomide-containing therapy and all had 2 prior lines of therapy, median PFS was 12.2 months and ORR was 32.1% [33]. In an interim analysis of cohort B, second- or third-line pomalidomide, daratumumab, and dexamethasone resulted in an ORR of 77.7% in a patient population that was 75.0% lenalidomide refractory and a 9-month PFS rate of 86.3% (range, 76.5–92.2%; median PFS was not reached) [34]. Immune analyses from the MM-014 trial demonstrated persistent T-cell–stimulatory activity (including an increase in CD8^+^ T cells without a decrease in CD4^+^ subsets) with pomalidomide-based regimens sequenced immediately after the failure of lenalidomide-based treatment, providing insight into how pomalidomide may overcome lenalidomide resistance [33, 35]. Moreover, the constituent agents of PVd have synergistic antimyeloma activity, and this combination stimulates T-cell proliferation despite the inhibitory effects of bortezomib [19].

Although caution must be used when evaluating results from multiple studies due to differences in study design and patient characteristics, the findings from this analysis add to the growing body of clinical trial data demonstrating the efficacy of pomalidomide-based triplet regimens in early-line RRMM. Patients in the phase 2 EMN011/HO114 trial received 8 cycles of pomalidomide, carfilzomib, and dexamethasone, followed by maintenance with pomalidomide alone or pomalidomide and dexamethasone, after having progressed on frontline lenalidomide- and bortezomib-based therapy during the phase 3 EMN02/HO95 trial; 95% of patients had PD during lenalidomide maintenance [36]. In an interim analysis, these patients achieved an ORR of 87% after 8 cycles of pomalidomide, carfilzomib, and dexamethasone. This same regimen resulted in an ORR of 84% in the phase 1/2 MMRC study (NCT01665794), which included lenalidomide-pretreated patients (91% lenalidomide refractory) in early-line RRMM (median 2 prior lines of therapy [range, 1–7 lines]) [37]. In a phase 2 trial of lenalidomide-exposed, nonrefractory patients, second-line treatment with pomalidomide, cyclophosphamide, and dexamethasone led to an ORR of 85% [38].

Other pomalidomide-based triplet therapies have been investigated in phase 3 trials that predominantly included lenalidomide-refractory patients with RRMM. The ICARIA study (NCT02990338) evaluated pomalidomide and dexamethasone with or without isatuximab in patients with ≥2 prior lines of therapy, including both lenalidomide and a proteasome inhibitor; the results of this study led to the approval of pomalidomide, dexamethasone, and isatuximab in this setting [24, 26]. Patients had a median of 3 prior lines of therapy (range, 2–11 lines), and 92.5% were refractory to lenalidomide in their last prior therapy. The median PFS was 11.5 months with the triplet regimen vs 6.5 months with the doublet (HR, 0.596 [95% CI, 0.44–0.81]; *P* = 0.001). ORR was also significantly higher with pomalidomide, dexamethasone, and isatuximab (60.4%) vs pomalidomide and dexamethasone (35.3%; *P* < 0.0001). We await results from the phase 3 APOLLO trial (NCT03180736), which is investigating pomalidomide, daratumumab, and dexamethasone in lenalidomide-refractory patients who have received ≥1 prior line of therapy (compared with the registrational MMY1001 phase 1b trial, in which patients had a median of 4 prior lines of therapy [range, 1–13 lines]) [39], including both lenalidomide and a proteasome inhibitor. Finally, although the phase 3 CANDOR trial (NCT03158688) did not evaluate a pomalidomide-based regimen, recent findings demonstrated a PFS benefit with carfilzomib, dexamethasone, and daratumumab vs carfilzomib and dexamethasone in both lenalidomide-exposed (39.4% vs 48.1% of patients; HR, 0.52 [95% CI, 0.34–0.80]) and lenalidomide-refractory patients (31.7% vs 35.7% of patients; HR, 0.45 [95% CI, 0.28–0.74]) [40].

In conclusion, the results of this subanalysis of the phase 3 OPTIMISMM study continue to demonstrate that PVd is effective in patients for whom lenalidomide is no longer a treatment option, including lenalidomide-refractory patients after 1 prior line of therapy. These findings also indicate that replacing an immunomodulatory agent with another drug class after lenalidomide treatment failure is not needed. The improvements in outcomes with PVd treatment across all subgroups of patients, including those with and without prior bortezomib exposure or SCT, demonstrate that pomalidomide-based regimens at first relapse confer benefits irrespective of previous exposure to these common frontline treatments.

## Data sharing

Data requests may be submitted to Celgene, a Bristol Myers Squibb Company, at https://vivli.org/ourmember/celgene/ and must include a description of the research proposal.

## Supplementary information

Supplemental Table 1

Supplemental Table 2

Supplemental Table 3

Supplemental Figure 1

Supplemental Figure 2

Supplemental Figure 3
