# Supplementary material for: Pomalidomide, bortezomib, and dexamethasone for multiple myeloma previously treated with lenalidomide (OPTIMISMM): outcomes by prior treatment at first relapse
Source: Leukemia. 2020 Sep 7;35(6):1722–31. doi: 10.1038/s41375-020-01021-3 (PMC8179841; doi:10.1038/s41375-020-01021-3)
Supplement: Supplementary file 1 — Supplemental Table 1 [file 41375_2020_1021_MOESM1_ESM.docx]

**Supplemental Table 1.** Baseline characteristics among patients at first relapse by prior exposure to bortezomib and prior stem cell transplant

| **Characteristic** | **Patients at first relapse^a^** | | | | | | | |
| --- | --- | --- | --- | --- | --- | --- | --- | --- |
|  | **Prior BORT** | | **No prior BORT** | | **Prior SCT** | | **No prior SCT** | |
|  | **PVd**  **(n = 67)** | **Vd**  **(n = 67)** | **PVd**  **(n = 44)** | **Vd**  **(n = 48)** | **PVd**  **(n = 56)** | **Vd**  **(n = 54)** | **PVd**  **(n = 55)** | **Vd**  **(n = 61)** |
| **Age, median (range), years**  > 65 years, n (%)  > 75 years, n (%) | 63 (29-78) 26 (38.8) 2 (3.0) | 64 (27-88) 30 (44.8) 8 (11.9) | 74 (51-87) 36 (81.8) 14 (31.8) | 70 (41-89) 34 (70.8) 10 (20.8) | 61 (38-73)  13 (23.2)  0 | 64 (41-78) 21 (38.9) 1 (1.9) | 73 (29-87) 49 (89.1) 16 (29.1) | 71 (27-89) 43 (70.5) 17 (27.9) |
| **Male, n (%)** | 39 (58.2) | 35 (52.2) | 28 (63.6) | 22 (45.8) | 35 (62.5) | 27 (50.0) | 32 (58.2) | 30 (49.2) |
| **ECOG PS, n (%)** |  |  |  |  |  |  |  |  |
| 0 | 43 (64.2) | 36 (53.7) | 24 (54.5) | 23 (47.9) | 40 (71.4) | 34 (63.0) | 27 (49.1) | 25 (41.0) |
| 1 | 23 (34.3) | 29 (43.3) | 18 (40.9) | 20 (41.7) | 15 (26.8) | 20 (37.0) | 26 (47.3) | 29 (47.5) |
| 2 | 1 (1.5) | 2 (3.0) | 2 (4.5) | 5 (10.4) | 1 (1.8) | 0 | 2 (3.6) | 7 (11.5) |
| **ISS stage, n (%)** |  |  |  |  |  |  |  |  |
| I | 45 (67.2) | 40 (59.7) | 20 (45.5) | 29 (60.4) | 36 (64.3) | 38 (70.4) | 29 (52.7) | 31 (50.8) |
| II | 17 (25.4) | 15 (22.4) | 16 (36.4) | 12 (25.0) | 15 (26.8) | 11 (20.4) | 18 (32.7) | 16 (26.2) |
| III | 5 (7.5) | 12 (17.9) | 8 (18.2) | 7 (14.6) | 5 (8.9) | 5 (9.3) | 8 (14.5) | 14 (23.0) |
| **Cytogenetic profile by FISH, n (%)^b^** |  |  |  |  |  |  |  |  |
| Standard risk | 32 (47.8) | 31 (46.3) | 26 (59.1) | 25 (52.1) | 27 (48.2) | 24 (44.4) | 31 (56.4) | 32 (52.5) |
| High risk | 11 (16.4) | 11 (16.4) | 7 (15.9) | 3 (6.3) | 11 (19.6) | 8 (14.8) | 7 (12.7) | 6 (9.8) |
| Missing or NE | 24 (35.8) | 25 (37.3) | 11 (25.0) | 20 (41.7) | 18 (32.1) | 22 (40.7) | 17 (30.9) | 23 (37.7) |
| **Time since MM diagnosis, median (range), years** | 3  (0.8-10.8) | 3.2  (0.6-12.8) | 3.0  (0.2-9.0) | 2.8  (0.4-11.1) | 3.1  (1.1-7.0) | 3.9  (1.0-7.2) | 2.9  (0.2-10.8) | 2.6  (0.4-12.8) |
| **Creatinine clearance < 60 mL/min, n (%)** | 12 (17.9) | 17 (25.4) | 23 (52.3) | 11 (22.9) | 4 (7.1) | 8 (14.8) | 31 (56.4) | 20 (32.8) |
| **Prior antimyeloma lines of therapy, median (range)** | 1 (1-1) | 1 (1-1) | 1 (1-1) | 1 (1-1) | 1 (1-1) | 1 (1-1) | 1 (1-1) | 1 (1-1) |
| **Previous treatment, n (%)** |  |  |  |  |  |  |  |  |
| Lenalidomide | 67 (100) | 67 (100) | 44 (100) | 48 (100) | 56 (100) | 54 (100) | 55 (100) | 61 (100) |
| Bortezomib | 67 (100) | 67 (100) | 0 | 0 | 49 (87.5) | 36 (66.7) | 18 (32.7) | 31 (50.8) |
| SCT | 49 (73.1) | 36 (53.7) | 7 (15.9) | 18 (37.5) | 56 (100) | 54 (100) | 0 | 0 |
| **Refractory status, n (%)^c^** |  |  |  |  |  |  |  |  |
| Lenalidomide | 36 (53.7) | 31 (46.3) | 28 (63.6) | 34 (70.8) | 26 (46.4) | 24 (44.4) | 38 (69.1) | 41 (67.2) |
| Bortezomib | 11 (16.4) | 7 (10.4) | 0 | 0 | 7 (12.5) | 0 | 4 (7.3) | 7 (11.5) |

BORT, bortezomib; ECOG PS, Eastern Cooperative Oncology performance status; FISH, fluorescence in situ hybridization; ISS, International Staging System; MM, multiple myeloma; NE, not evaluable; PD, progressive disease; PVd, pomalidomide, bortezomib, and dexamethasone; SCT, stem cell transplant; Vd, bortezomib plus dexamethasone.

^a^ Patients with only 1 prior line of therapy. ^b^ High risk was defined as presence of ≥ 1 of the following cytogenetic abnormalities: del(17p) (including monosomy 17), t(4;14), and/or t(14;16). Standard risk was defined as the absence of high-risk cytogenetic abnormalities. ^c^ Refractory disease was defined as disease that was nonresponsive to treatment (failure to achieve minimum response or development of PD) within 60 days of the last dose, inclusive.
