# Supplementary material for: Pomalidomide, bortezomib, and dexamethasone for multiple myeloma previously treated with lenalidomide (OPTIMISMM): outcomes by prior treatment at first relapse
Source: Leukemia. 2020 Sep 7;35(6):1722–31. doi: 10.1038/s41375-020-01021-3 (PMC8179841; doi:10.1038/s41375-020-01021-3)
Supplement: Supplementary file 2 — Supplemental Table 2 [file 41375_2020_1021_MOESM2_ESM.docx]

**Supplemental Table 2.** Overall response rate in patients at first relapse

| **Response rate, n (%)** | **Patients at first relapse^a^** | |
| --- | --- | --- |
|  | **PVd**  **(n = 111)** | **Vd**  **(n = 115)** |
| **Overall response rate** | 100 (90.1) | 63 (54.8) |
| ≥ VGPR | 68 (61.3) | 26 (22.6) |
| sCR | 6 (5.4) | 2 (1.7) |
| CR | 14 (12.6) | 5 (4.3) |
| VGPR | 48 (43.2) | 19 (16.5) |
| PR | 32 (28.8) | 37 (32.2) |
| **SD** | 10 (9.0) | 40 (34.8) |
| **PD** | 1 (0.9) | 4 (3.5) |
| **NE** | 0 | 8 (7.0) |

CR, complete response; NE, not evaluable; PD, progressive disease; PR, partial response; PVd, pomalidomide, bortezomib, and dexamethasone; sCR, stringent complete response; SD, stable disease; Vd, bortezomib plus dexamethasone; VGPR, very good partial response.

^a^ Patients with only 1 prior line of therapy.
