# Supplementary material for: Pomalidomide, bortezomib, and dexamethasone for multiple myeloma previously treated with lenalidomide (OPTIMISMM): outcomes by prior treatment at first relapse
Source: Leukemia. 2020 Sep 7;35(6):1722–31. doi: 10.1038/s41375-020-01021-3 (PMC8179841; doi:10.1038/s41375-020-01021-3)
Supplement: Supplementary file 3 — Supplemental Table 3 [file 41375_2020_1021_MOESM3_ESM.docx]

**Supplemental Table 3.** Overall response rate among patients at first relapse by prior bortezomib exposure and prior SCT

| **Response rate, n (%)** | **Patients at first relapse^a^** | | | | | | | | | | |
| --- | --- | --- | --- | --- | --- | --- | --- | --- | --- | --- | --- |
|  | **Prior BORT** | | **No prior BORT** | | | | **Prior SCT** | | **No prior SCT** | | |
|  | **PVd**  **(n = 67)** | **Vd**  **(n = 67)** | | **PVd**  **(n = 44)** | **Vd**  **(n = 48)** | **PVd**  **(n = 56)** | | **Vd**  **(n = 54)** | | **PVd**  **(n = 55)** | **Vd**  **(n = 61)** |
| Overall response rate | 60 (89.6) | 33 (49.3) | | 40 (90.9) | 30 (62.5) | 51 (91.1) | | 31 (57.4) | | 49 (89.1) | 32 (52.5) |
| ≥ VGPR | 42 (62.7) | 11 (16.4) | | 26 (59.1) | 15 (31.3) | 39 (69.6) | | 11 (20.4) | | 29 (52.7) | 15 (24.6) |
| sCR | 3 (4.5) | 0 | | 3 (6.8) | 2 (4.2) | 5 (8.9) | | 1 (1.9) | | 1 (1.8) | 1 (1.6) |
| CR | 10 (14.9) | 2 (3.0) | | 4 (9.1) | 3 (6.3) | 7 (12.5) | | 2 (3.7) | | 7 (12.7) | 3 (4.9) |
| VGPR | 29 (43.3) | 9 (13.4) | | 19 (43.2) | 10 (20.8) | 27 (48.2) | | 8 (14.8) | | 21 (38.2) | 11 (18.0) |
| PR | 18 (26.9) | 22 (32.8) | | 14 (31.8) | 15 (31.3) | 12 (21.4) | | 20 (37.0) | | 20 (36.4) | 17 (27.9) |
| SD | 6 (9.0) | 25 (37.3) | | 4 (9.1) | 15 (31.3) | 4 (7.1) | | 18 (33.3) | | 6 (10.9) | 22 (36.1) |
| PD | 1 (1.5) | 3 (4.5) | | 0 | 1 (2.1) | 1 (1.8) | | 2 (3.7) | | 0 | 2 (3.3) |
| NE | 0 | 6 (9.0) | | 0 | 2 (4.2) | 0 | | 3 (5.6) | | 0 | 5 (8.2) |

BORT, bortezomib; CR, complete response; NE, not evaluable; PD, progressive disease; PR, partial response; PVd, pomalidomide, bortezomib, and dexamethasone; sCR, stringent complete response; SCT, stem cell transplant; SD, stable disease; Vd, bortezomib plus dexamethasone; VGPR, very good partial response.

^a^ Patients with only 1 prior line of therapy.
