# Supplementary figures and images for: Pomalidomide, bortezomib, and dexamethasone for multiple myeloma previously treated with lenalidomide (OPTIMISMM): outcomes by prior treatment at first relapse
Source: Leukemia. 2020 Sep 7;35(6):1722–31. doi: 10.1038/s41375-020-01021-3 (PMC8179841; doi:10.1038/s41375-020-01021-3)

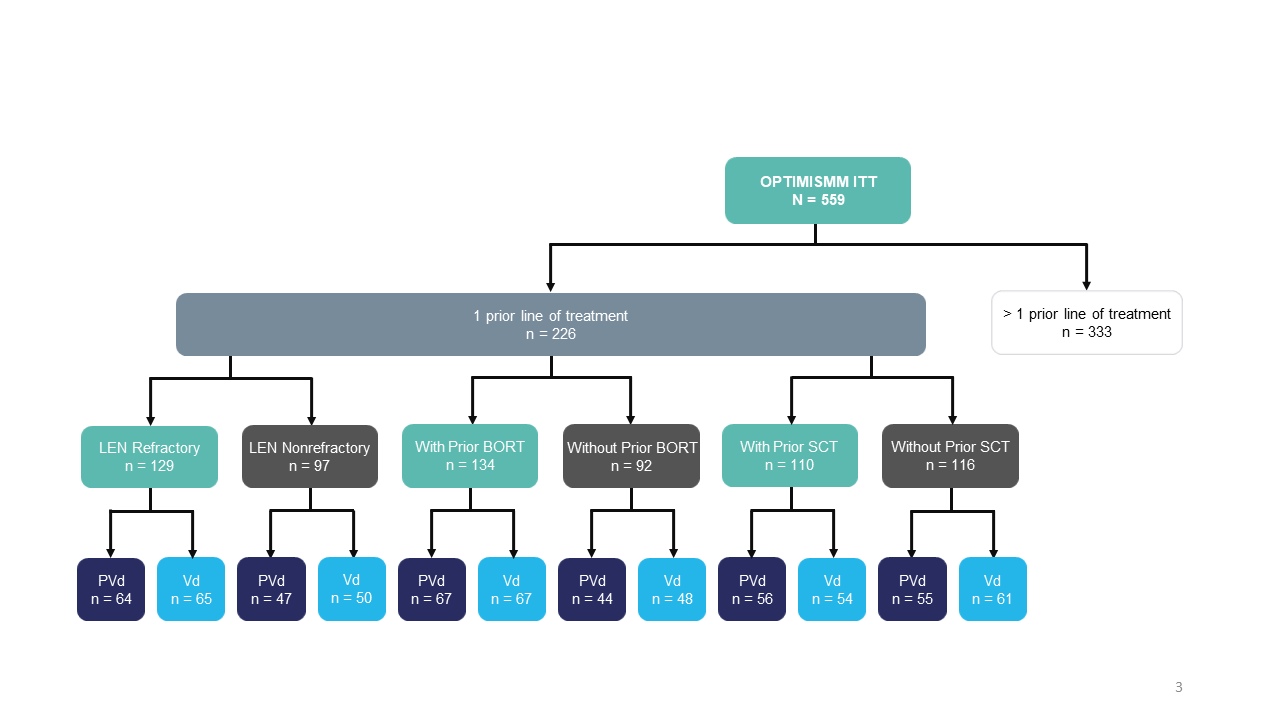

Supplement: Supplementary file 4 — Supplemental Figure 1 [file 41375_2020_1021_MOESM4_ESM.tif]

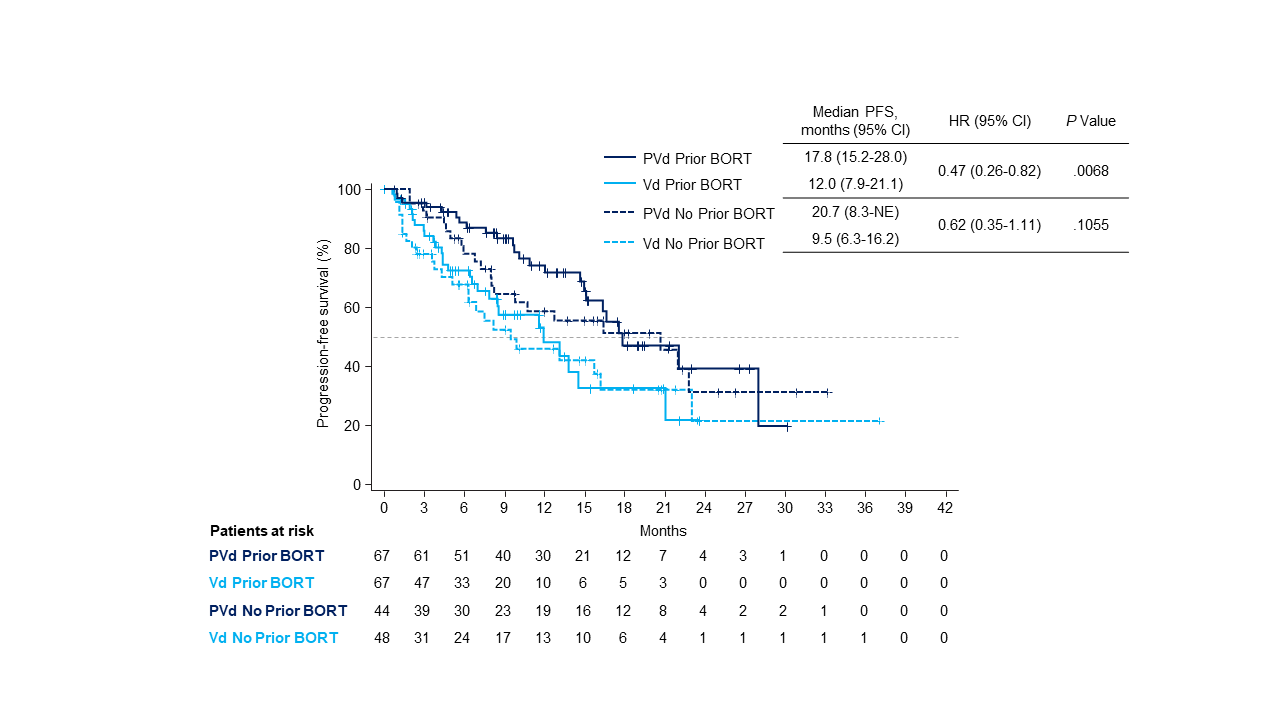

Supplement: Supplementary file 5 — Supplemental Figure 2 [file 41375_2020_1021_MOESM5_ESM.tif]

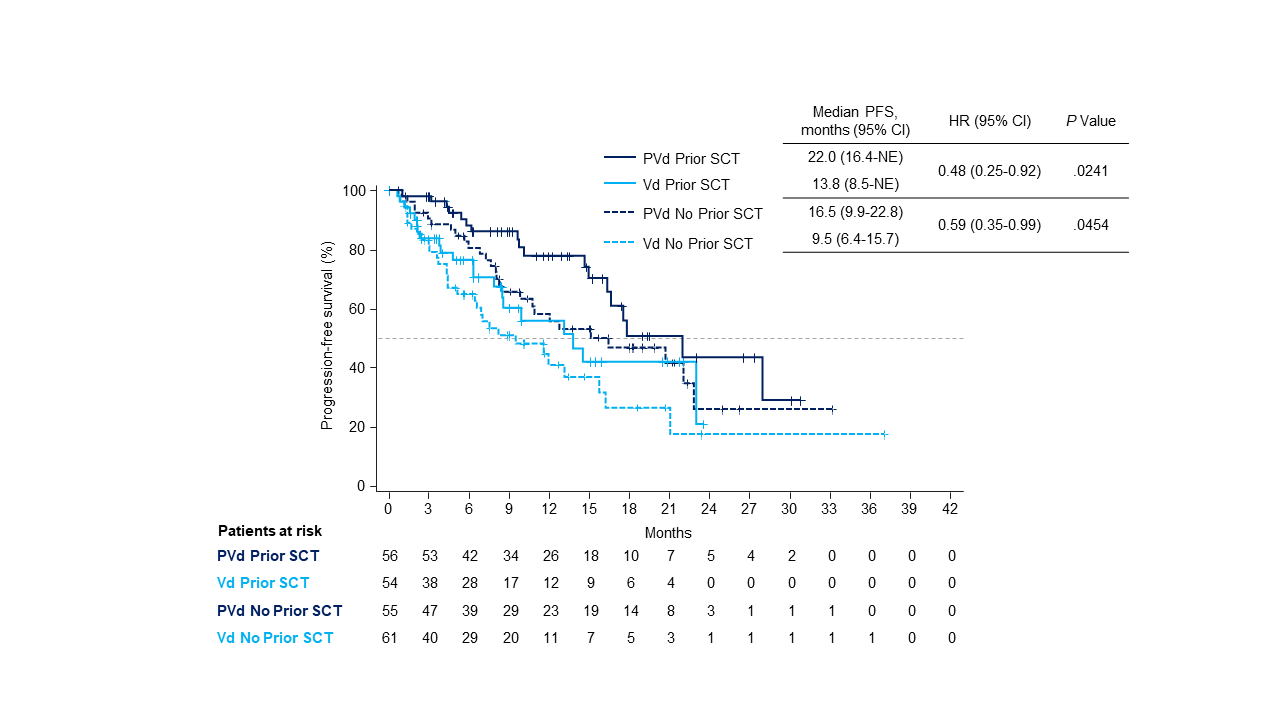

Supplement: Supplementary file 6 — Supplemental Figure 3 [file 41375_2020_1021_MOESM6_ESM.tif]
